# A Rare Presentation of Systemic Sclerosis

**DOI:** 10.7759/cureus.48599

**Published:** 2023-11-10

**Authors:** Joud Enabi, Maha Tahir, Srikanth Mukkera, Alejandra Garcia Fernandez

**Affiliations:** 1 Internal Medicine, Texas Tech University Health Sciences Center, Odessa, USA; 2 Rheumatology, Texas Tech University Health Sciences Center at Permian Basin, Odessa, USA; 3 Internal Medicine/Intensive Care Unit, Texas Tech University Health Sciences Center, Odessa, USA

**Keywords:** aortic regurgitation, systemic scleroderma, autoimmune disease, rheumatology, systemic sclerosis

## Abstract

Systemic sclerosis (SSc) is a persistent autoimmune disorder. While it commonly impacts the cardiac valves, particularly the mitral valve, involvement of the aortic valve has been seldom documented. We report a case of a 47-year-old male with a history of progressive SSc who displayed complications related to cardiac issues, which were verified through a left atrial appendage biopsy revealing thickening due to fibrosis. This cardiac involvement led to a condition necessitating the replacement of the aortic valve due to aortic regurgitation. This instance underscores the importance of identifying this uncommon association, enabling the delivery of appropriate patient treatment, and reducing complications linked to the underlying condition.

## Introduction

Systemic sclerosis (SSc) is a complex chronic disease characterized by widespread microvascular injury and progressive skin and organ fibrosis. The new classification criteria for SSc, established by the ACR-EULAR committee, employed a multi-criteria point system. The classification requires skin thickening of the fingers extending proximal to the MCPs as sufficient to be classified as Ssc. However, if absent, manifestations such as skin thickening of the fingers, fingertip lesions, telangiectasia, abnormal nail fold capillaries, interstitial lung disease or pulmonary arterial hypertension, Raynaud's phenomenon, and SSc-related autoantibodies are required [1]. Although not included in the criteria for diagnosis, cardiac involvement is common but often unrecognized until the disease's advanced stages; involvement impacts the myocardium, pericardium, and conduction system. Although valvular heart disease (VHD) was previously considered rare in SSc, recent studies show elevated risks of aortic stenosis (AS), aortic regurgitation (AR), and mitral regurgitation (MR) in SSc patients compared to the general population [2]. Patients diagnosed with SSc exhibit an approximately five-fold higher likelihood of experiencing moderate to severe mitral and aortic valvular disease (MAVD) when contrasted with individuals without SSc (odds ratio [OR] 4.60). The prevailing anomaly is MR, trailed by AS and AR [3]. Notably, evident cardiac complications in SSc are linked to unfavorable outcomes, increasing mortality risk.

## Case presentation

**Figure 1:**
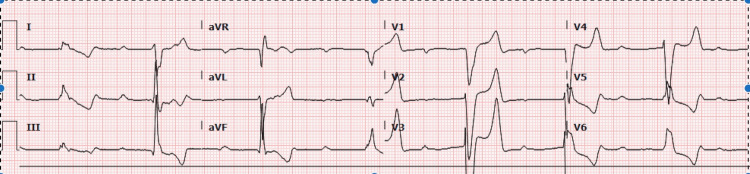
An electrocardiogram (ECG) demonstrated a complete heart block with a left bundle branch block and elevated troponin levels

A 47-year-old male with a complex rheumatologic history, including rheumatoid arthritis, systemic lupus erythematosus, Raynaud's phenomenon, SSc with diffuse cutaneous sclerosis, interstitial pulmonary fibrosis, AVNRT (atrioventricular nodal reentrant tachycardia) status post-ablation, and pericarditis, presented with acute onset loss of consciousness due to a complete heart block and shock with associated renal failure. On the point of contact, the patient's vital signs revealed hypotension (63/30 mmHg) and severe bradycardia (heart rate of 43 bpm). Laboratory tests showed increased serum creatinine (3.2 mg/dL), blood urea nitrogen (BUN) (30 mg/dL), and lactate levels (6.04 mmol/L). An electrocardiogram (ECG) demonstrated a complete heart block with a left bundle branch block and elevated troponin levels (Figure 1). The patient's blood pressure improved with vasopressor support and intravenous fluids, and lactate levels normalized after resuscitation.

**Figure 2:**
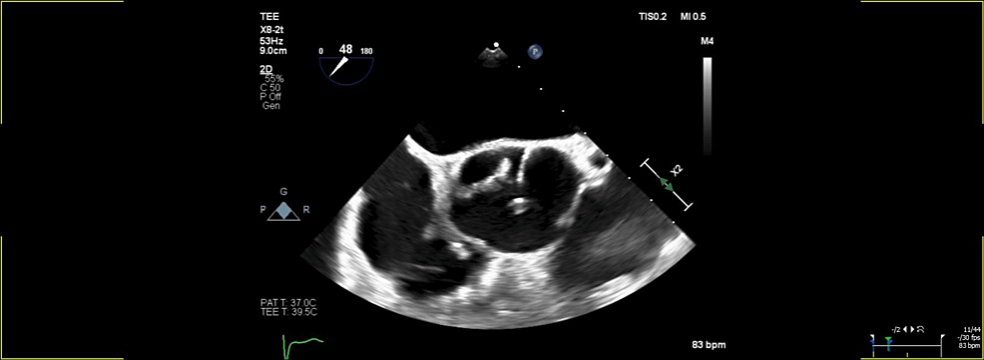
Echocardiogram showing severe AR, prompting consideration of valve replacement AR: Aortic regurgitation

Moreover, the patient experienced recurrent episodes of heart block overnight, which were exacerbated by massive hemoptysis leading to acute respiratory failure, necessitating intubation and proning therapy, with no evidence of diffuse alveolar hemorrhage found during bronchoalveolar lavage. Subsequent transesophageal echocardiography confirmed severe AR, prompting consideration of valve replacement (Figure 2). Additionally, the patient developed digital cyanotic changes. Aortic valve replacement was performed using a standard 23 mm On-X mechanical prosthesis, and a bovine patch was used to repair the right coronary cusp annulus abscess. The left atrial appendage biopsy revealed benign characteristics, with mild fibrosis indicating cardiac involvement in the underlying SSc. Elevated ANA titers and scleroderma antibody IgG further supported the diagnosis. The myocardial tissue displayed minimal chronic inflammation with scattered lymphocytes, while no definitive ischemic changes were observed. Notably, the pericardial tissue showed no evidence of fibrous pericarditis or adhesions. These biopsy findings collectively suggest a cardiac manifestation of SSc without significant malignancy or vasculopathy, highlighting the importance of managing the disease's impact on the heart. For those without SSc, with a prevalence almost five times greater (OR 4.60), MR is the most common lesion, followed by AS and AR. On the other hand, the development of mitral stenosis is less frequent [2]. 

## Discussion

SSc is a disease that affects multiple systems and is characterized by damage to small blood vessels and progressive fibrosis of the skin and internal organs. The heart is commonly affected but may go undetected until later in the disease. All heart parts, including the myocardium, pericardium, and conduction system, can be affected [1]. Cardiac involvement often indicates a poor prognosis and a higher risk of death. The comprehensive exploration of VHD within the context of SSc has yet to be undertaken. The existing data primarily stem from pathological investigations that delineate changes reminiscent of endocarditis on valves such as mitral, tricuspid, or aortic valves observed during postmortem examinations [2]. While instances of AR and mitral valve prolapse arising from the thickening of the aortic and mitral valves have been documented before, no methodical examination of VHD within a population-based incident cohort-specific to SSc has been reported to the best of our knowledge [3]. The prevailing valvular involvement most often encountered in SSc is tricuspid regurgitation, frequently associated with pulmonary hypertension, although not exclusively so [4].

A previous Danish nationwide cohort study reported a roughly three-fold increase in the risk of AS, a four-fold increase in the incidence of AR, and a higher risk of mitral increased relative risk of MR five times in patients with SSc compared to the healthy population [5]. Increased screening echocardiography might explain this increased prevalence, or it could be due to SSc-specific factors that may contribute to its development. Another case-control study investigating the prevalence of MAVD in patients with SSc revealed that SSc patients have a significantly higher chance of experiencing moderate to severe MAVD compared to those without SSc, with a prevalence almost five times greater (OR 4.60) with MR being the most common lesion followed by AS and AR. On the other hand, the development of mitral stenosis is less frequent [6]. Aortic incompetence was documented with lesser frequency in the available literature. Narváez et al. observed a notably higher occurrence of AR in individuals with SSc compared to control subjects [6]. Additionally, Fernández-Codina et al. established a significant connection between AR and the limited skin subset of SSc [7]. Notably, there appears to be a lack of information regarding pulmonary valve insufficiency in SSc within the existing literature [8].

## Conclusions

Cardiac complications are a common manifestation of systemic sclerosis, and this case accentuates the importance of recognizing and managing them. In cases of severe cardiac involvement, interventions such as valve replacements may be prompted. Vigilant cardiac monitoring, punctual intervention, and comprehensive management are crucial for optimizing outcomes in patients with complex rheumatologic histories and systemic sclerosis-related cardiac manifestations.
